# Stochastic gradient descent for optimization for nuclear systems

**DOI:** 10.1038/s41598-023-32112-7

**Published:** 2023-05-25

**Authors:** Austin Williams, Noah Walton, Austin Maryanski, Sandra Bogetic, Wes Hines, Vladimir Sobes

**Affiliations:** Nuclear Engineering, University of Tennessee, Knoxville, 37996 USA

**Keywords:** Engineering, Nuclear fusion and fission

## Abstract

The use of gradient descent methods for optimizing k-eigenvalue nuclear systems has been shown to be useful in the past, but the use of k-eigenvalue gradients have proved computationally challenging due to their stochastic nature. ADAM is a gradient descent method that accounts for gradients with a stochastic nature. This analysis uses challenge problems constructed to verify if ADAM is a suitable tool to optimize k-eigenvalue nuclear systems. ADAM is able to successfully optimize nuclear systems using the gradients of k-eigenvalue problems despite their stochastic nature and uncertainty. Furthermore, it is clearly demonstrated that low-compute time, high-variance estimates of the gradient lead to better performance in the optimization challenge problems tested here.

## Introduction

Autonomous algorithmic-based design is becoming a powerful optimization tool in many engineering fields^1,2^. Nuclear engineering stands to gain from these powerful optimization tools. These automated tools can help explore vast design spaces where the optimal case is neither intuitive nor easy to find due to an intractable number of potential solutions. Automated design of nuclear systems can also help break away from traditional human biases that are often present. For example, an AI system chose to add a moderator material within a region that was intended to be a “fast spectrum” section for shaping a neutron energy spectrum of the Fast Neutron Source^3^ at the University of Tennessee, Knoxville.

Gradient descent^4,5^ has been shown to be an effective tool for engineering problems^6–8^. Nuclear engineering has little documentation on the use of these methods within the field^9–12^. The autonomous design algorithm used in this work is a stochastic gradient descent. This algorithm utilizes the derivatives of an objective function to iteratively take steps toward the minimum of the objective function and is designed to work well with a stochastic gradient, such as those estimated by Monte Carlo neutron transport simulations. Previously, this group published the demonstration of gradient descent for the design optimization of neutronic systems with quantitative constraints using the interior point method to optimize the k-eigenvalue, $k_{eff}$, and reaction rates^13^. The major issue brought up by the previous work was the stochastic nature of the gradients built from Monte Carlo neutron transport simulations. In this work, the gradient is built from sensitivities estimated using the Tools for Sensitivity and Uncertainty Analysis Methodology Implementation, TSUNAMI, code from the SCALE package^14–16^. Sensitivity in this analysis describes how sensitive an eigenvalue or reaction rate ratio is to the total macroscopic cross-section, $\Sigma$. The sensitivities are used as the gradient in the form shown in Eq. (1), where f is either $k_{eff}$ or reaction rate based on the problem.

1 $$\begin{aligned} S=\frac{df}{d\Sigma } \end{aligned}$$

## Methodology

In this section, the methods used to implement the gradient descent model ADAM coupled with TSUNAMI are discussed. Also, the challenge problems used to test if ADAM is a suitable optimization method for nuclear processes are introduced.

### Algorithm

The gradient descent model used in this analysis is the ADAM (Adaptive moment estimation) method^17^. The ADAM method name stands for adaptive moment estimate. ADAM combines two well-established gradient descent methods: Adagrad^18^ and RMSProp^19^. ADAM leverages Adagrad’s ability to use sparse gradients and RMSProp’s utility with on-line and non-stationary settings. ADAM is designed to ensure step size magnitude does not change with gradient changes, works well with sparse gradients, and has natural step size annealing. The ADAM algorithm can be seen below, where $\beta _{1}$, $\beta _{2}$, $\upepsilon$ and $\upalpha$ are hyperparameters used to tune performance, *t* is the current step, *x* is the list of variables being optimized, $g_{t}$ is the gradient vector, *m* is the first-moment estimate, *v* is the raw second-moment estimate, $\hat{m}$ is the bias-corrected first-moment estimate, and $\hat{v}$ is the bias-corrected second-moment estimate. The variables $\beta _{1}$ and $\beta _{2}$ are limited on [0, 1) and determine the momentum of the algorithm, $\upalpha$ adjusts the step size of the algorithm, and $\upepsilon$ is used to ensure the algorithm is not dividing by zero. Momentum, in this case, refers to a gradient’s likelihood to stay on its current path.

2 $$\begin{aligned} m_{t}&=\beta _{1}m_{t-1}+(1-\beta _{1} )g_{t} \end{aligned}$$

3 $$\begin{aligned} v_{t}&=\beta _{2}v_{t-1}+(1-\beta _{2} )g_{t}^{2} \end{aligned}$$

4 $$\begin{aligned} \vec {m_{t}}&=\frac{m_{t}}{1-\beta _{1}^{t}} \end{aligned}$$

5 $$\begin{aligned} \vec {v_{t}}&=\frac{v_{t}}{1-\beta _{2}^{t}} \end{aligned}$$

6 $$\begin{aligned} x_{t}&=x_{t-1}-\frac{\alpha \vec {m_{t}}}{\sqrt{\vec {v_{t}}}+\varepsilon } \end{aligned}$$

### Gradient

The gradients for this work are calculated using TSUNAMI, a Monte Carlo based method, which makes them stochastic by nature. This means that the derivatives informing the gradient descent algorithm are noisy and have an associated uncertainty. The previously used Interior Point Method does not account for uncertainty or noise, meaning the gradient was assumed to be exact. This requires long TSUNAMI run times or the resulting gradients are noisy. The use of ADAM intends to correct this issue due to the built-in accounting for uncertainty within the gradient. This allows for high-variance sensitivities to be used to optimize nuclear systems.

The continuous energy version TSUNAMI-3D is used to calculate eigenvalue and reaction sensitivities for constructing the gradients used in the analysis. This version of TSUNAMI-3D provides 2 methods to calculate the sensitivity of k-eigenvalue: Iterative Fission Probability (IFP) and Contribution-Linked eigenvalue sensitivity/Uncertainty estimation via Tracklength importance CHaracterization (CLUTCH). Both methods use general perturbation theory to calculate the first-order sensitivity values^14^. The IFP method calculates the adjoint-weighted tallies and the importance for future generations, based on the neutron population. The CLUTCH method uses an importance function and determines sensitivity through the number of fission neutrons created by a collision^20^. The IFP method is used for the optimization of $k_{eff}$ because it requires fewer neutron histories to be used and therefore allows us to use very high-variance sensitivities with fast run times to build the gradient for each step.

The sensitivity of the reaction rate is calculated using general perturbation theory through the GEneralized Adjoint Responses in Monte Carlo (GEAR-MC) method which uses both the CLUTCH and IFP methods^21^. This method calculates the generalized importance function as a sum of intergenerational (IFP) and intragenerational (CLUTCH) effects. The sensitivities for both $k_{eff}$ and reaction rate are valid to use as gradients for this analysis because they are an unbiased estimator of the gradient^14^. Sensitivities are defined as functions of the macroscopic cross-section of the material. The sensitivity with respect to the macroscopic cross-section can be used as a density sensitivity because the macroscopic cross-section is the product of material number density and microscopic cross-section. In this work, microscopic cross sections and molar mass are assumed to be known for a given material, resulting in the relationship, shown in Eq. (2), between macroscopic cross section and the mass density of the material:

7 $$\begin{aligned} \Sigma =N\sigma =\frac{N_{A}\rho \sigma }{M} \end{aligned}$$

where *N* is the number density, $\upsigma$ is the total microscopic cross section, $N_{A}$ is Avogadro’s number, *M* is the molar mass, and $\uprho$ is the mass density of a material—the physical design parameter that is varied for optimization.

## Results

### Proposal of challenge problems

Four challenge problems are proposed to test the use of TSUNAMI to build a gradient for the ADAM method. All four challenge problems are expected to have a smooth gradient (when the sensitivities have very low statistical variance) and no local minima other than the global minima. Challenge problems one, two, and three mirror the challenge problems developed in the first publication^13^. The set-up is a 55 cm $\times$ 55 cm 2-dimensional, unreflected system. This geometry is then discretized into pixels, with the material being constant within each pixel. The material for each pixel is allowed to be a fixed homogenous mixture of $UO_{2}$ (at 3% enrichment) and $H_{2}O$. The density of this mixture in each pixel is varied as a parameter for optimization. This mixture is a ratio of 1 $UO_{2}$ to 3.4 $H_{2}O$. The SCALE material card is reported in the additional information section. This problem is purposefully designed to have a known optimal $k_{eff}$ of 1 with a perfectly circular geometry. The pixelated version of the optimal solution has a $k_{eff}$ slightly less than 1, depending on the spatial resolution. These problems aim to test ADAM:

1. as a nuclear system optimization algorithm,
2. test if a constraint can be implemented into the gradient, and
3. determine if high-variance sensitivities can be used in gradient descent optimization.

In the first challenge problem, the prism is broken into an $11 \times 11$ grid of 5 cm $\times$ 5 cm pixels that each have a unique density. The density in each pixel is expressed by the Sigmoid function, defined as *f*(*x*) in Eq. (3), which is used to allow *x* to be in the range of negative infinity to positive infinity while restricting the density to remain in the range of zero to one. For this challenge problem, zero represents void and unity represents the density of a homogenized light-water-reactor fuel pin. This challenge problem optimizes the density of each pixel to maximize $k_{eff}$ with a constraint on the total mass of the system. The amount of total mass of the system is restricted to the mass of 61 pixels of the nominal density. This value was chosen as 61 because the system can become critical with 61 pixels (50.4% full) of the material in a cylindrical configuration. This problem aims to test the ability of ADAM to maximize the performance of a nuclear challenge problem while the variables are constrained.

8 $$\begin{aligned} f(x)=\frac{1}{1+e^{-x}} \end{aligned}$$

To enforce the constraint on the $k_{eff}$ optimization problem, the objective function is changed such that the score is lowered if the mass goes above 61 by an exponential penalty term. This method goes against the norm within the community of using the log-barrier method^22,23^ because we wanted to allow the simulation to violate the constraint during the course of the optimization. The hyperparameters of this exponential penalty term need to be optimized such that they force the mass to the desired constraint. The equation used for the objective function and gradient for this problem can be seen below. The function, *O*(*x*), is the optimization function and $\frac{dO}{dx}$ is the gradient used within the ADAM algorithm. The variables *r* and *v* are parameters that allow the penalty function to be tuned such that it only takes effect once the constraint is exceeded and *S*(*x*) represents the sensitivities calculated by TSUNAMI.

9 $$\begin{aligned} O(\vec {x})&=re^{v(\sum _{i=1}^{121}(f(x_{i}))-61)}-k_{eff}(\vec {x}) \end{aligned}$$

10 $$\begin{aligned} \frac{dO}{dx_{i}}&=re^{v(\sum _{i=1}^{121}(f(x_{i}))-61)}v\frac{e^{-x_{i}}}{(1+e^{-x_{i}})^{2}}-S(x_{i}) \end{aligned}$$

The second challenge problem uses the same geometry and density variable as problem one but aims to minimize mass with a constraint on $k_{eff}$. The constraint is set such that $k_{eff}$ must be greater than unity. This problem demonstrates the use of TSUNAMI sensitivities in the constraint function. A new objective function was developed to minimize the mass of the system while constraining the $k_{eff}$ of the system. The new equation used can be seen below where *O*(*x*) is the objective function, $\frac{dO}{dx}$ is the gradient used within ADAM, *r* is a tuning parameter for the $k_{eff}$ constraint, *x* is the set of one hundred twenty-one parameters for mass, and *S*(*x*) refer to the sensitivities pulled from TSUNAMI.

11 $$\begin{aligned} O(\vec {x})&=re^{v(1-k_{eff} (\vec {x}))} -\sum _{i=1}^{121}f(-x_{i}) \end{aligned}$$

12 $$\begin{aligned} \frac{dO}{dx_{i}}&=-re^{v(1-k_{eff}(\vec {x}))}vS(x_{i})-\frac{e^{-x_{i}}}{(1+e^{-x_{i}})^{2} } \end{aligned}$$

The third challenge problem is an expansion of the $11 \times 11$ geometry. This problem mimics challenge problem one’s geometry with a $44 \times 44$ pixelation where the outer dimensions are still 55 cm $\times$ 55 cm, and the material is varied in a similar way. The same material is used for this problem as the previous problems. The number of full cells is changed proportionally to ensure the same amount of material is used. The new number of cells used as the mass constraint is $\frac{61\times 44^{2}}{11^{2}}=976$. This is the only change to the objective function and derivatives used in the first challenge problem, where 61 is replaced with 976. This problem aims to show that when we expand the number of variables within the system, the ADAM algorithm can still converge. The finer grid also gives ADAM more geometric freedom to form a better-resolved solution. It should also be noted that the sensitivities from TSUNAMI will have a larger relative uncertainty per pixel due to the finer spacial discretization. Therefore, challenge problem three will show how an increase in uncertainty and noise in the derivatives will not affect ADAM’s ability to find a solution.

The fourth challenge problem is an 80 cm slab geometry reflected (symmetric) on two axes, effectively creating a 1-dimensional problem. This slab is then divided into 8 equal regions in the non-reflected direction. The geometry is also reflected on the face of region one, doubling the slab size with material symmetry. The slab is made of the same material as the previous problem. This geometry was chosen to represent the axial flux shape of a 1-dimensional system. The objective of this challenge problem is to flatten the fission reaction rate profile across all cells by changing the density of the material in each region. This problem aims to test the ability of the GPT method of reaction rate sensitivity as a gradient for optimization. Below are the equations used for the objective function and derivatives used for this challenge problem, where *i* and *j* refer to the discretization locations, *RR* refers to the reaction rate, and $S(x_{i})$ is the sensitivity of the reaction rate ratio at location *i* over location *j*.

13 $$\begin{aligned} O(\vec {x})&=\sum _{i=1}^{8}\sum _{j=1}^{8}\left( 1-\frac{RR_{i}(\vec {x})}{RR_j (\vec {x})}\right) ^{2} \end{aligned}$$

14 $$\begin{aligned} \frac{dO}{dx_{i}}&=\sum _{j=1}^{8}\left( \frac{2}{RR_j}\left( \frac{RR_{i}}{RR_j}-1\right) S(x_{i})\right) \end{aligned}$$

The implementation of ADAM, for the challenge problems solved in this article, utilizes sensitivities from TSUNAMI as the gradient directly. TSUNAMI outputs sensitivities in two ways: material-based and element-based. For the challenge problems chosen, material sensitivities are used, because the problems are not optimizing the ratio of the material. They are optimizing the location of the material. The TSUNAMI run made at each step is constructed to use very little computation time to test if the algorithm works with a high-variance gradient. Each TSUNAMI run uses 10 skipped generations, 5 latent generations, 10 active generations, and 10,000 neutrons per generation, which allows us to use these short calculations, and high-variance gradients.

### Verification of challenge problems

**Figure 1:**
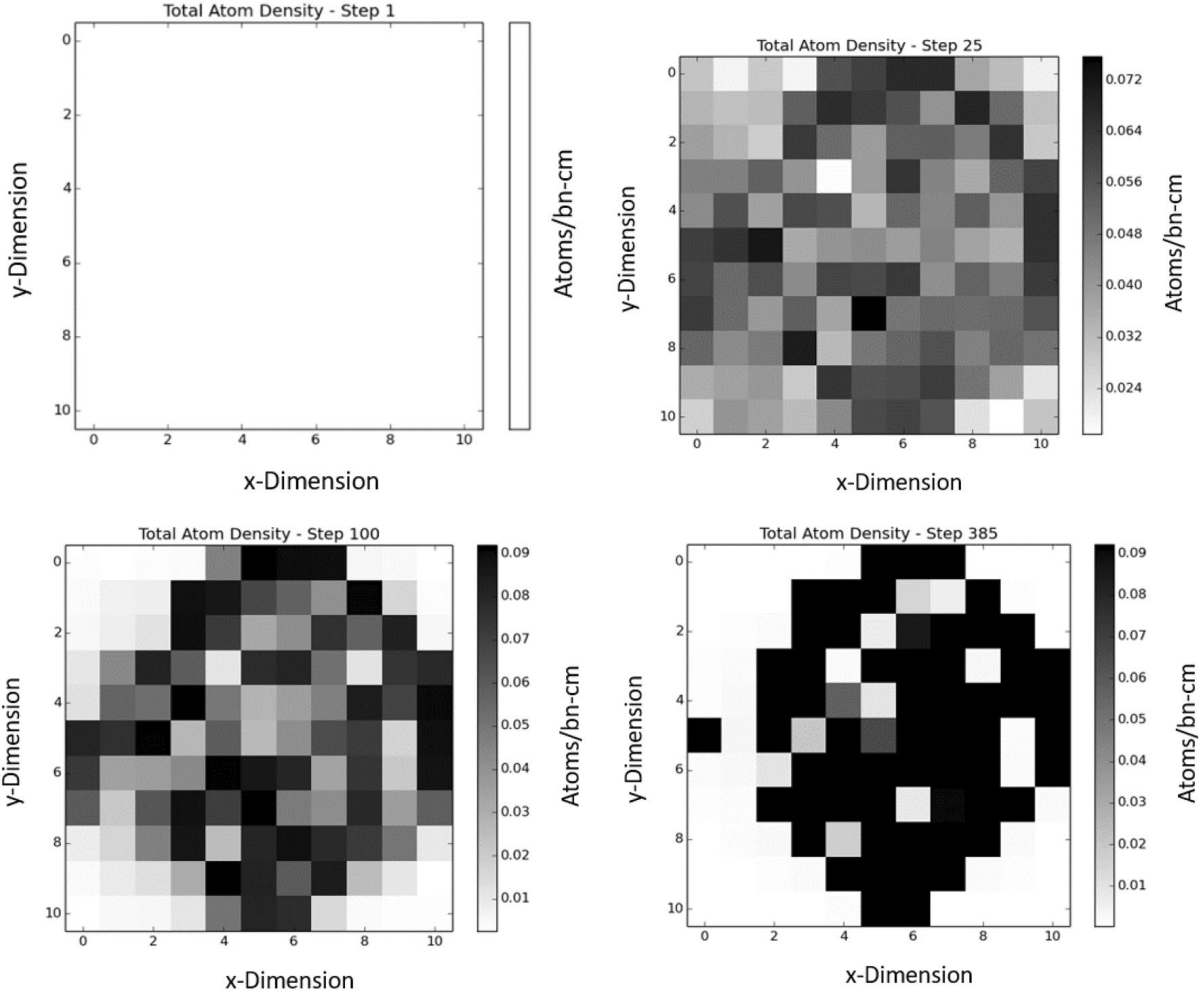
This figure shows the movement of material throughout the course of optimization of an $11 \times 11$ cell geometry. The algorithm optimized the geometry to maximize $k_{eff}$ with a constraint on the mass within the system. The steps show how the geometry moves from a flat geometry toward a cylindrical shape.

**Figure 2:**
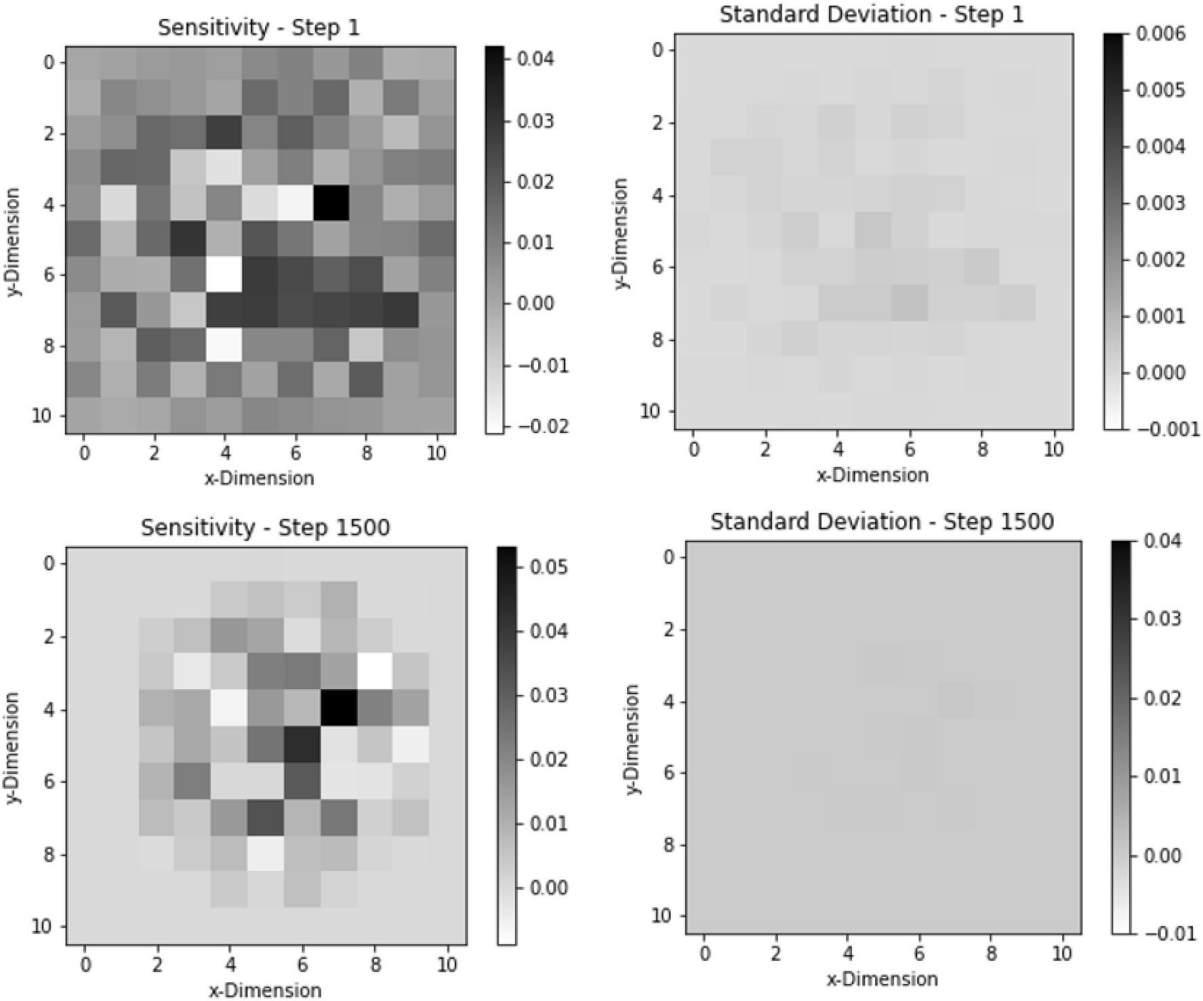
The sensitivity (with standard deviation) for the first and final are shown here. This shows that the sensitivities do not change much throughout the course of optimization. This helps ADAM to maintain on-line approximation of the derivatives for the problem.

Challenge problem one is an $11 \times 11$ discretization of a $55 \times 55$ cm prism reflected in the vertical direction. Each pixel has an associated density that ranges from 0 to the nominal density of the homogenized fuel pin. This problem aims to determine if the TSUNAMI sensitivities can be used as a gradient for optimization. ADAM uses the derivatives of the objective function presented in the “Methodology” section to optimize the material density (effectively managing the location of the material) within the system. An innovative aspect of this approach already discussed in previous work^13^ is that setting the density to be a continuous variable is one way to approach the design optimization of arbitrary geometry. A penalty term can be added to force the density to converge to either 0 or unity at the end of the optimization creating a solution with discrete density. In this case, the physics of the problem force the optimal solution to converge to 0/1 density for all pixels. The initial condition of the system begins with the material evenly distributed over all cells with the mass being slightly less than the constraint where $k_{eff}$ is below 0.75. ADAM is able to optimize the location of the material to find a $k_{eff}$ of $0.9806 \pm 0.0032$. The change in material location can be seen in Fig. 1 below. This shows how the material was moved into a cylindrical shape, which is optimal for this system. Note that the optimal solution is symmetric as all pixelated versions of the cylinder with 61 units of mass will have the same calculated $k_{eff}$ regardless of rotation or location. Figure 2 shows the sensitivity profiles in the first and last steps of the algorithm. The sensitivities’ average magnitude is $0.00507 \pm 0.00362$. This shows that ADAM can be used to optimize the $k_{eff}$ for a system with high-variance sensitivities because the sensitivities have 71% uncertainty on average.

**Figure 3:**
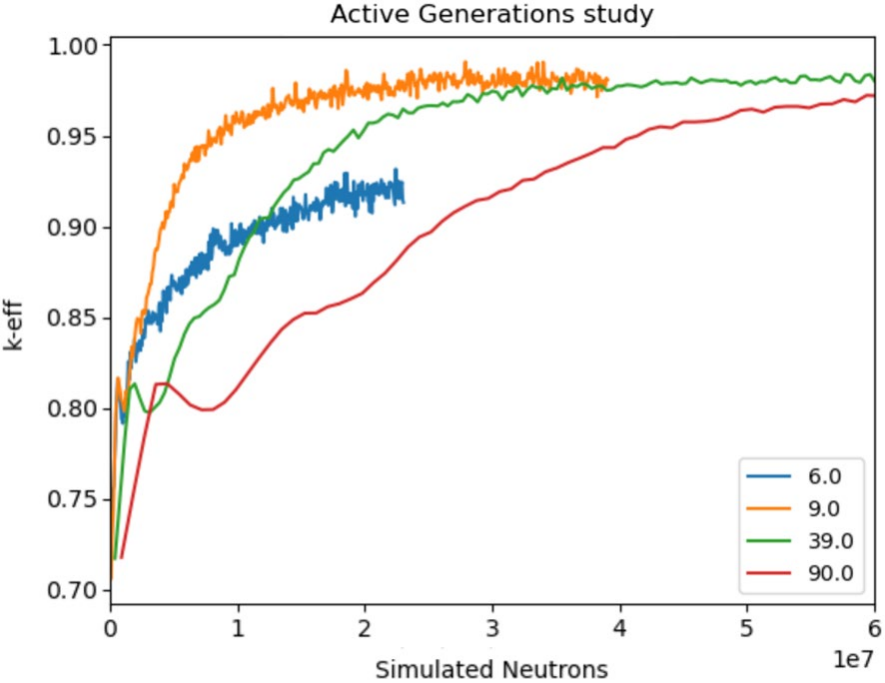
This shows the effect of running different numbers of simulated neutrons per step for the k-eigenvalue optimization with a fixed $\upalpha$ value of 0.1. Each different colored line shows a different number of simulated neutrons where the x-axis is the total number of neutrons simulated. This shows that running different numbers of simulated neutrons per step can have a dramatic effect on the total amount of neutrons needed to reach convergence. Do note that the number of simulated neutrons is directly proportional to runtime.

**Figure 4:**
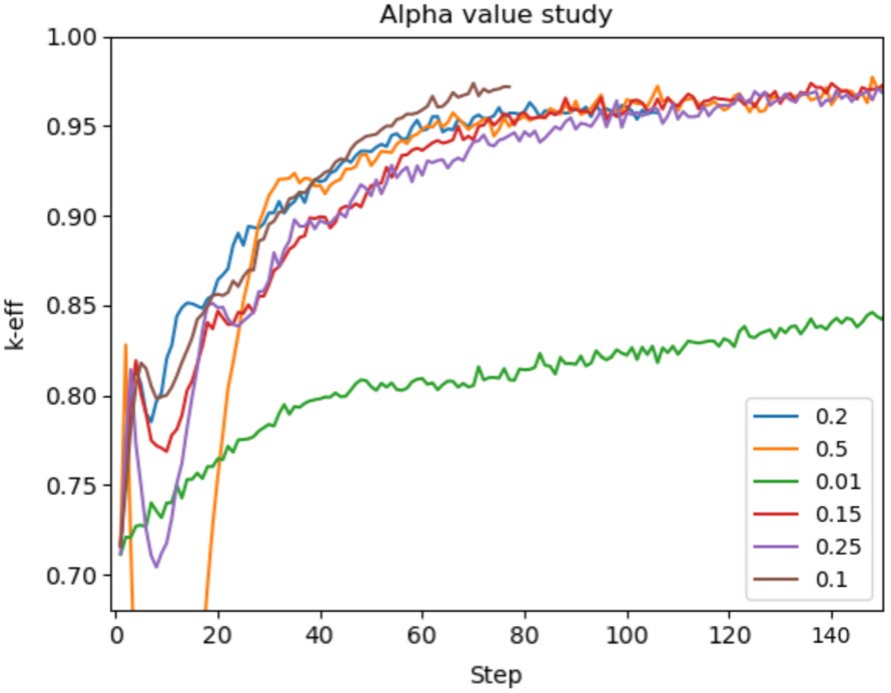
This plot shows the effects of changing the $\upalpha$ hyperparameters after a different number of steps within ADAM with a constant number of 10 simulated neutrons per step. Each line represents a different variation of $\upalpha$. This demonstrates that $\upalpha$ can have an effect on the stability and number of steps needed to converge. $\upalpha$ must be fine-tuned to receive the best results.

The main parameters used to tune the performance of ADAM for this problem are the number of simulated neutrons per update step and the step size hyperparameter $\upalpha$. Figures 3 and 4 show different results while isolating the tuning of these parameters. The number of simulated neutrons is proportional to the computational runtime for each step and the statistical accuracy of the derivatives. Although more simulated neutrons produce more lower-variance, better performance was seen with many, quick, high-variance steps rather than with few, long, low-variance steps. This is shown in Fig. 3 by plotting the approach to optimal of $k_{eff}$ against the cumulative number of neutrons simulated. All of the plots seem to approach the same asymptotic behavior where lower neutrons per step (lower runtime) are favorable. Do note that the case of 6 generations is an exception to this trend. The $\upalpha$ analysis (Fig. 4) shows that a larger value can help to speed up the approach to optimal $k_{eff}$, but too large a value of $\upalpha$ can be volatile as seen in the case where $\alpha = 0.5$. Alternatively, the lowest value of $\upalpha$ (0.05) approaches the optimal with fewer depressions but increased the time needed to reach the maximum.

Challenge problem 2 is the same geometry as challenge problem 1. This problem aims to test the ability to use TSUNAMI sensitivities as part of the constraint for the optimization. ADAM uses the derivatives of the objective function presented in the “Methodology” section to optimize the mass within the system by changing pixel densities within the system.

**Figure 5:**
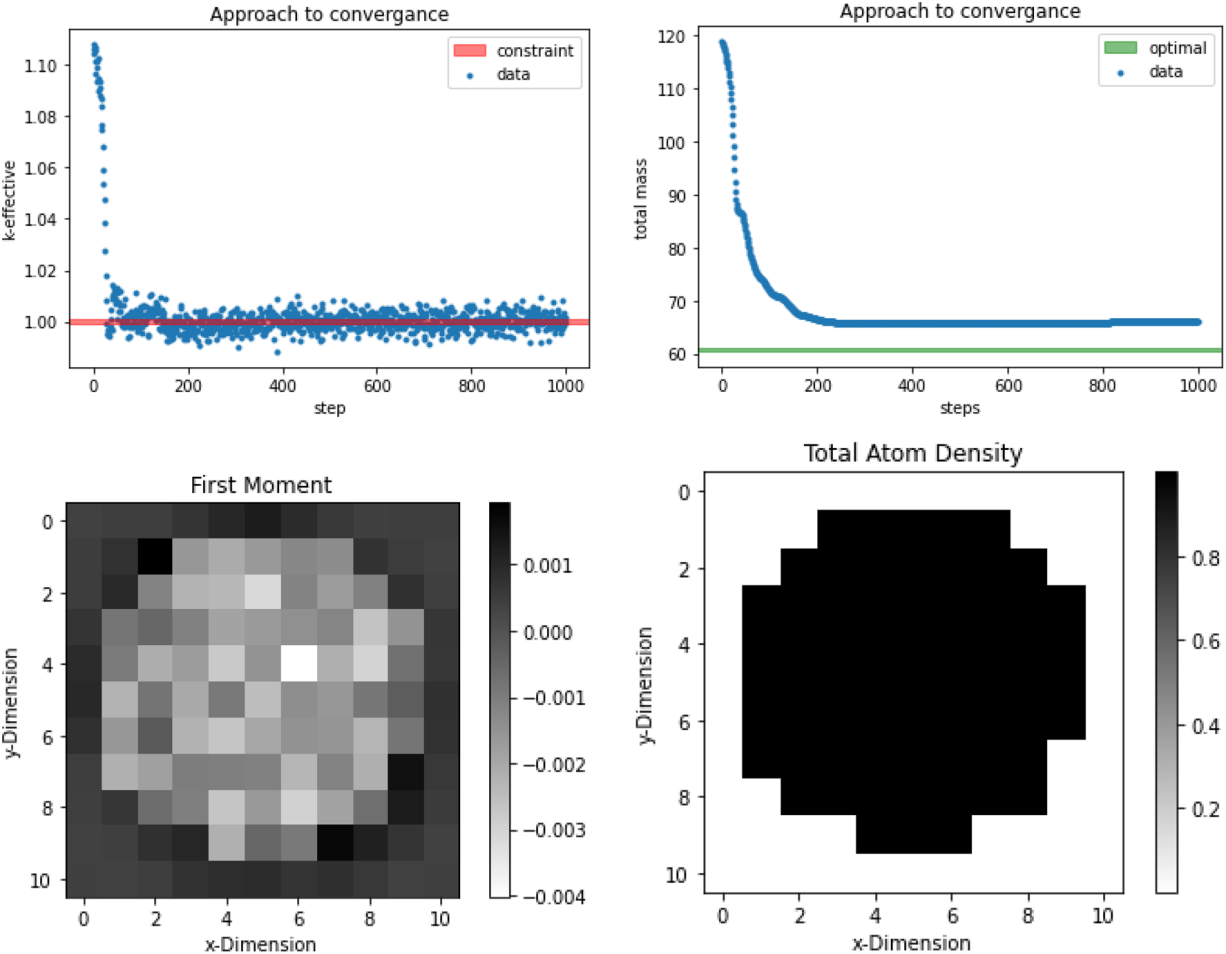
The top-left plot shows the $k_{eff}$ throughout the course of the $11 \times 11$ $k_{eff}$ optimization. The top-right plot shows the mass of the system throughout the optimization. ADAM was reach the optimal value of $k_{eff}$ but not within the constraint of mass. This was expected because the optimization was constructed to start with a large mass and would descend to an optimal. The bottom-left plot shows the first moment from Eq. (4) for the best case found. This shows that ADAM was removing material from the outside and maintaining mass near the center. The bottom-right figure shows the mass of the system for the best case. This shows that ADAM was able to use the first moment to design an optimal design.

The ADAM algorithm was able to maintain a $k_{eff}$ of 1.0018 with a mass of 65.95 units. The approach to this result can be seen in Fig. 5, below. This result was due to the algorithm getting stuck in local minima due to the coarse nature of the $11 \times 11$ pixelation. The resulting configuration has the expected shape, similar to challenge problem 1, for the optimal result, as seen in Fig. 5.

**Figure 6:**
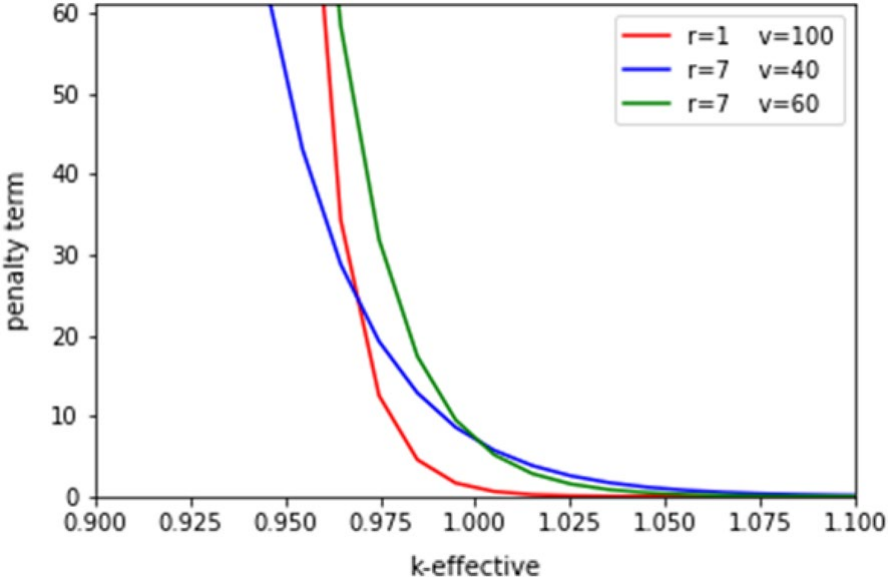
The different hyperparameters of the penalty term are shown with differing values. These hyperparameters must be tuned to reach an optimal value but can be precomputed to be close to the best configuration.

The hyperparameters in the penalty term required tuning for this challenge problem. Figure 6, below, shows different tunings of constraint hyperparameters, r and v. These show different cases where the penalty term can begin to drop off very sharply once the constraint is met or can take time to fall off even after the constraint is met. This tuning can be used to enforce a strict result where the constraint is always met or allow for results where the constraint is close to being met but the system mass is lower.

Challenge problem 3 is a $44 \times 44$ discretization of the 55 cm $\times$ 55 cm prism reflected on the vertical axis. Each pixel has an associated density that ranges from zero to the nominal density. This problem aims to determine whether higher-variance derivatives and a larger optimization space will improve or hinder the ability of the ADAM algorithm. ADAM uses the derivatives of the objective function presented in the “Methodology” section to optimize the mass within the system by changing pixel densities within the system.

**Figure 7:**
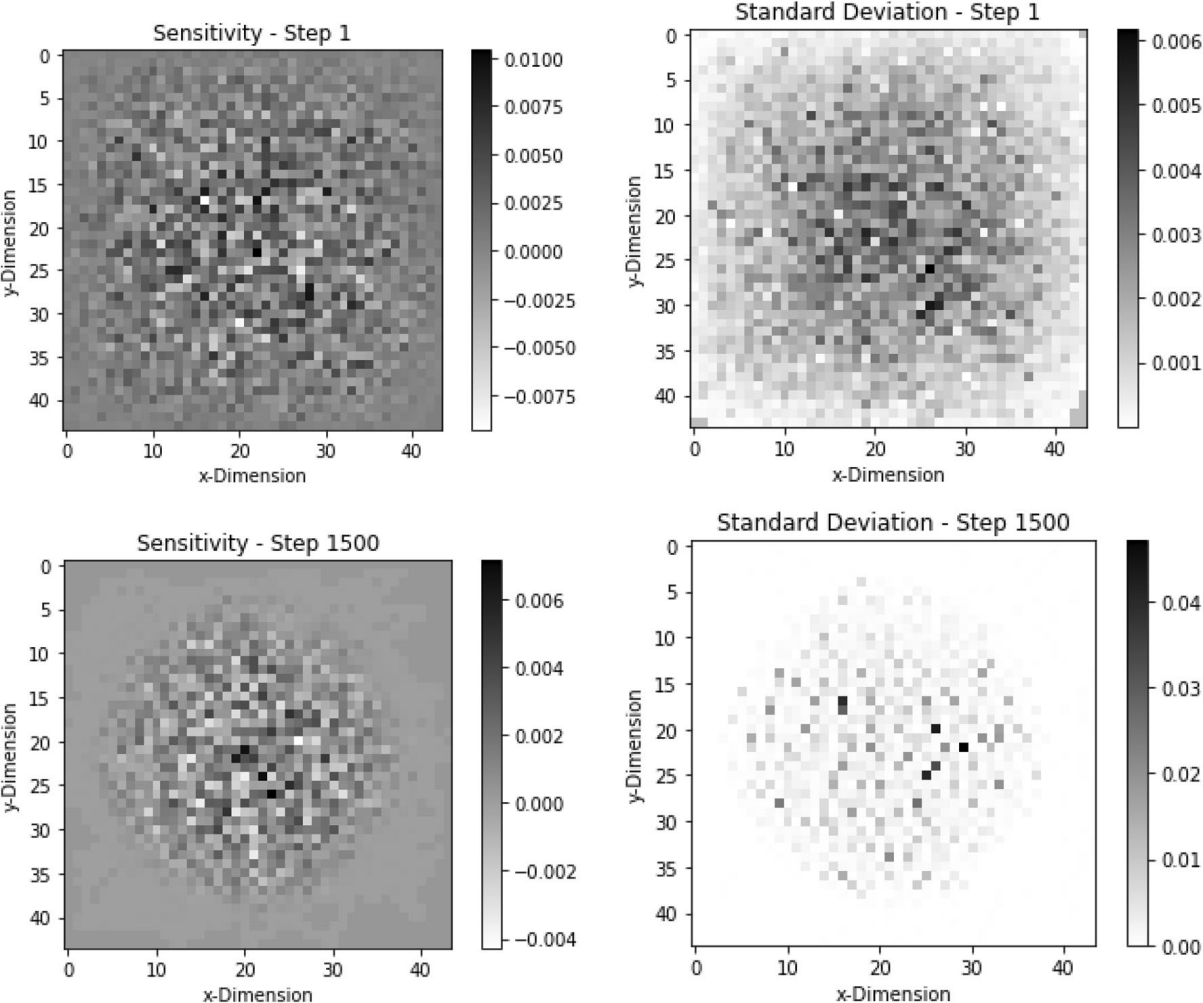
This figure shows the sensitivities and standard deviation for the first and last step of the $44 \times 44$ geometry optimization of $k_{eff}$. The sensitivity started out with a random sensitivity profile but converged to a cylindrical shape once the material was moved toward the middle of the geometry. The standard deviations follow the same trend but remain sparse throughout the optimization.

The sensitivity magnitudes of the problem are $0.000537 \pm 0.000599$ on average. The sensitivity profiles for the first and last step of this convergence can be seen above in Fig. 7. It is also important to note that many sensitivity values have uncertainty greater than 100%. The first step has 1086 out of 1936 sensitivity values where uncertainty was greater than 100%. This result further supports the case that ADAM does not require converged sensitivity values to reach a converged result, rather it prefers to take many quick steps at the cost of higher uncertainty on the sensitivities. The solution also shows that ADAM will perform better with more geometric options than with better gradient convergence.

**Figure 8:**
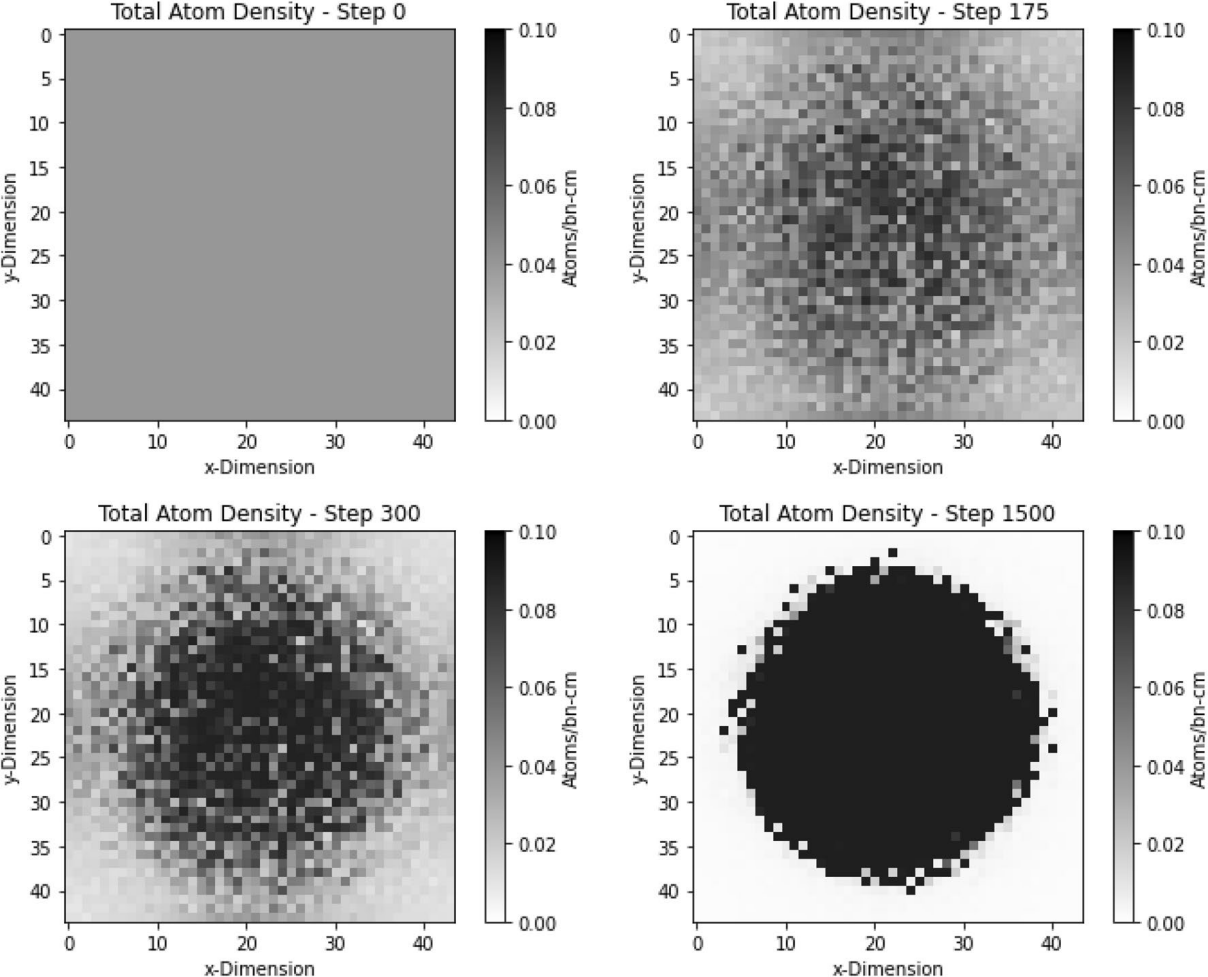
These figures show the material movement throughout the course of the $44 \times 44$ $k_{eff}$ optimization. It can be seen that the material moves toward the center of the geometry. The finer mesh of the $44 \times 44$ geometry allows for the shape to be more cylindrical.

**Figure 9:**
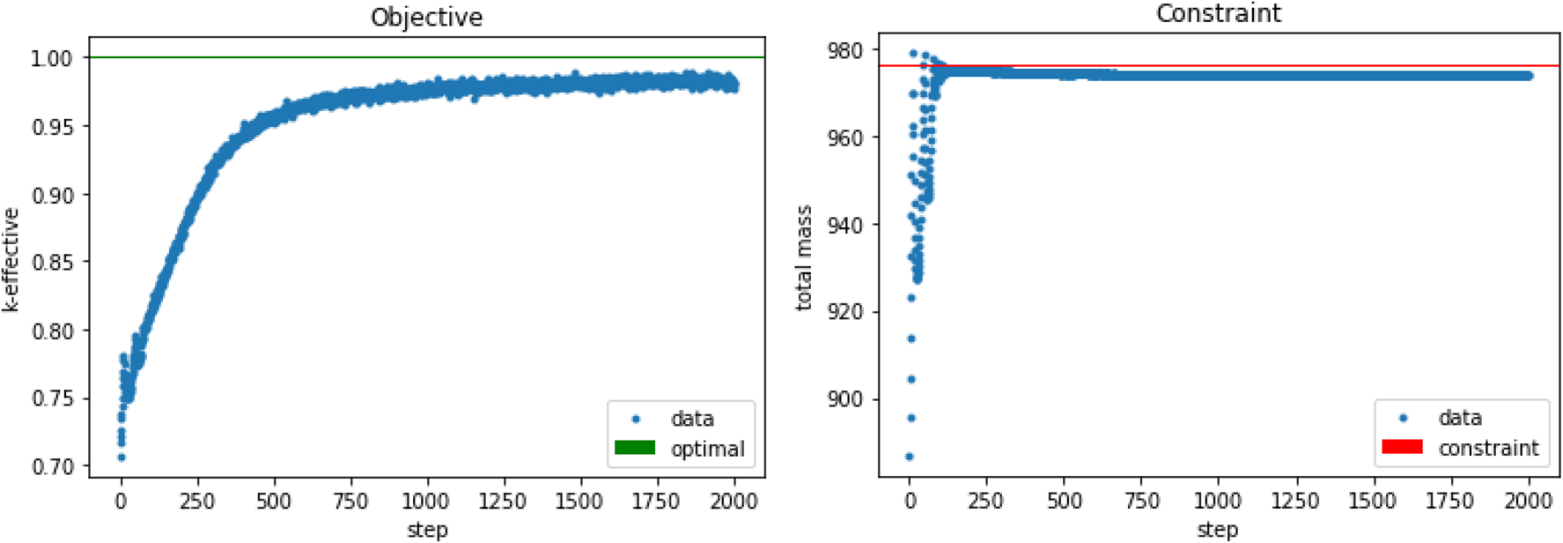
The left image shows the optimization of $k_{eff}$ for the $44 \times 44$ geometry. The right image shows the mass constraint over the course of the optimization. These show that the optimal value was not reached because the mass constraint was maintained.

The $44 \times 44$ geometry problem ended on a $k_{eff}$ of 0.98910 with a total of 973.9 fuel cell mass after 2000 steps. This is greater than the $k_{eff}$ of the $11 \times 11$ problem while maintaining the mass constraint. Figure 8, above, shows how the mass moved within the system over the course of the algorithm. Figure 9 shows ADAM’s approach to the chosen $k_{eff}$ constraint. Figure 9, also, shows the mass constraint of the problem over each step. With a finer mesh grid, the material was allowed to form a cylindrical design, which is optimal for this system. The percentage uncertainty on the sensitivities is, on average, 1.56$\times$ larger for each cell in the $44 \times 44$ system than the $11 \times 11$ system. This shows that even though the ADAM algorithm had higher-variance sensitivities for each parameter, it could still find a better solution to the problem presented.

**Figure 10:**
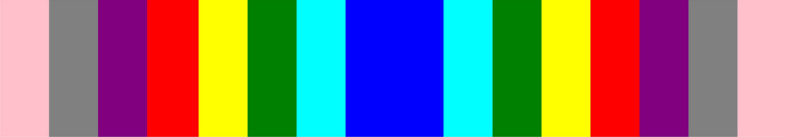
This figure shows the slab geometry used for challenge problem 4. Each different color slab represents a different cell with its own mass. Notice the symmetry across the middle of the slab. This representation is used to visualize the reflective boundary condtion of the slab.

Challenge problem 4 was designed to test the ability of ADAM to use reaction rate sensitivity from TSUNAMI as a gradient. The geometry for the system is a 1D slab geometry divided into eight 10-cm regions with reflective boundaries on the face of region one, as seen in Fig. 10. The objective of this problem is to optimize the fission reaction rate profile to be flat. The parameters for this optimization are the density variable of each cell. The gradients used for this problem are the fission reaction rate ratio sensitivities calculated using TSUNAMI’s GEAR-MC method.

**Figure 11:**
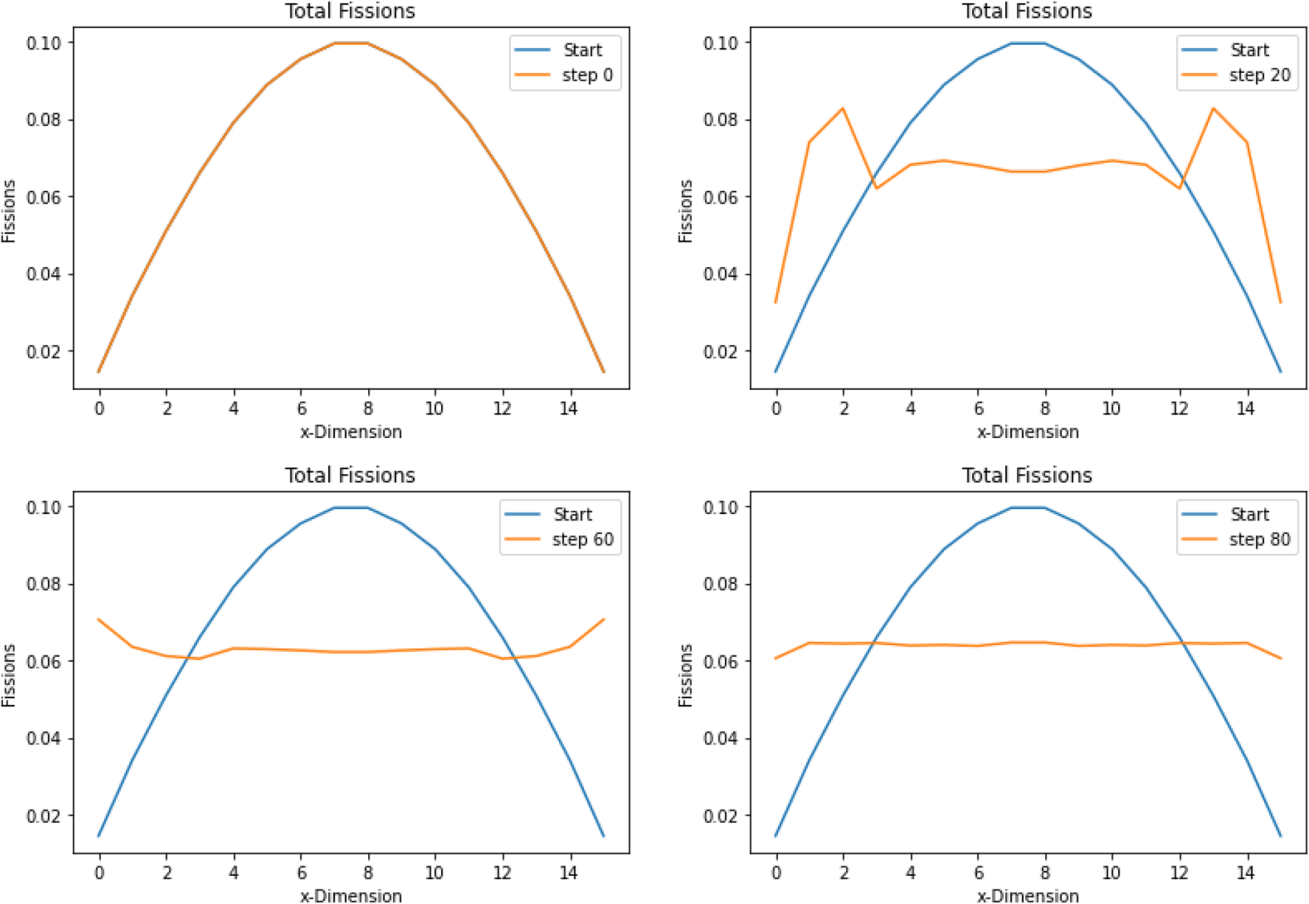
This shows the fission reaction rate in each slab throughout the optimization. The optimization was to flatten the fission rate across the slab geometry. This visualization shows the fission reaction rates flattening at the final step.

**Figure 12:**
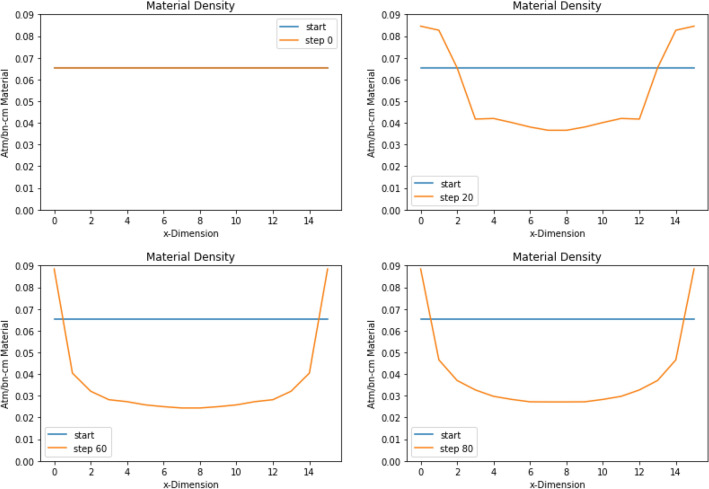
This figure shows how the material is moved throughout the optimization to flatten the fission reaction rates. It can be seen how the material is pushed to the side where the reaction rates where lower initially. This geometry was able to achieve a flat fission rate for this problem.

The cells begin with evenly distributed material. This results in a sinusoidal flux shape as seen in Fig. 11, with the fission reaction rate ranging from 0.02 to 0.1. ADAM was able to use the reaction rate sensitivities to find a system where the fission reaction rates across all cells ranged from 0.060 to 0.065. Figure 11 shows how the fission rate changes throughout the algorithm. It also shows the final step taken compared to the initial case. This shows visually how the fission reaction rate is flattened across the system. Figure 12 shows how ADAM adjusted the material within the system to find the optimal fission reaction rate.

## Discussion

In conclusion, ADAM and TSUNAMI work well together for optimizing $k_{eff}$ problems despite the stochastic nature of the gradient. The first two challenge problems demonstrated the use of high-variance $k_{eff}$ sensitivities from TSUNAMI for the gradient of the objective function and the penalty term, respectively. The first problem was able to find a $k_{eff}$ of $0.9806 \pm 0.0032$ with a mass below the constraint of 61 units. Problem two was able to reach a mass of 65.95 with a $k_{eff}$ above the constraint of criticality ($k_{eff} > 1.00$). Challenge problem 3 displayed that ADAM scales excellently with increasing dimensionality of the search space and gives a better answer with a finer geometry structure despite the associated uncertainty increase. The key demonstration was that with the ADAM method, it is most efficient to use very high-variance sensitivity information. The final $k_{eff}$ value was 0.98910 with 50.3% of a full geometry. The fourth challenge problem demonstrated the use of reaction rate ratio sensitivity from TSUNAMI as the gradient of ADAM. The problem was able to reduce the reaction rate ratio from 80 to 7.7%. These problems demonstrated the validity of using high-variance TSUNAMI $k_{eff}$ and reaction rate sensitivities for $k_{eff}$ problems. ADAM was able to use these high-variance gradients and reach solutions that match the desired outcomes.These results show that ADAM could potentially be used to optimize nuclear systems dominated by neutronics, such as searching for a maximum reactivity configuration in criticality safety applications.

## Supplementary Information

Supplementary Information.

## Data Availability

The data sets generated and analyzed during the current study are available in the ADAM repository, https://github.com/Naww137/ADAM.
