# Supplementary material for: Stochastic gradient descent for optimization for nuclear systems
Source: Sci Rep. 2023 May 25;13:8474. doi: 10.1038/s41598-023-32112-7 (PMC10213052; doi:10.1038/s41598-023-32112-7)
Supplement: Supplementary file 1 — Supplementary Information. [file 41598_2023_32112_MOESM1_ESM.docx]

**Appendix:**

MCNP:

c density 0.073272 molecules/cc

M1 092234 9.303759E-7

092235 1.006667E-4

092236 3.721503E-7

092238 0.003212

008016 0.027739

001001 0.042219

SCALE:

u-234 1 0 9.303758558e-07 300 end

u-235 1 0 1.006666676e-04 300 end

u-236 1 0 3.721503423e-07 300 end

u-238 1 0 0.0032122157 300 end

o-16 1 0 0.0277388212 300 end

h-1 1 0 0.0422190049 300 end
